# Prognosticators of survival in patients with metastatic pancreatic cancer and ascites

**DOI:** 10.1016/j.esmoop.2023.102048

**Published:** 2023-11-16

**Authors:** J.M. Berger, A. Alany, L. Berchtold, R. Puhr, A. Friedrich, B. Scheiner, G.W. Prager, M. Preusser, A.S. Berghoff, E.S. Bergen

**Affiliations:** 1 Division of Oncology, Department of Medicine I, Medical University of Vienna, Vienna; 2 Christian Doppler Laboratory for Personalized Immunotherapy, Department of Medicine I, Medical University of Vienna, Vienna; 3 Division of Gastroenterology and Hepatology, Department of Medicine III, Medical University of Vienna, Vienna, Austria

**Keywords:** ascites, metastatic pancreatic cancer, liver metastases, peritoneal carcinomatosis, systemic inflammation

## Abstract

**Background:**

Identification of factors associated with survival after ascites diagnosis in metastatic pancreatic cancer (mPC) patients may guide treatment decisions and help to maintain quality of life in this highly symptomatic patient collective.

**Patients and methods:**

All patients treated for mPC at the Medical University of Vienna between 2010 and 2019 developing ascites throughout their course of disease were identified by retrospective chart review. General risk factors, metastatic sites, systemic inflammation and liver function parameters, as well as type of treatment after ascites diagnosis were investigated for associations with survival.

**Results:**

One hundred and seventeen mPC patients with ascites were included in this study. Median time from mPC to ascites diagnosis was 8.9 months (range 0-99 months) and median overall survival (OS) after ascites diagnosis was 27.4 days (range 21.3-42.6 days). Identified prognostic factors at ascites diagnosis independently associated with an impaired OS were presence of liver metastases [hazard ratio (HR): 2.07, 95% confidence interval (CI) 1.13-3.79, *P* = 0.018), peritoneal carcinomatosis (HR: 1.74, 95% CI 1.11-2.71, *P* = 0.015), and portal vein obstruction (HR: 2.52, 95% CI 1.29-4.90, *P* = 0.007). Compared with best supportive care, continuation of systemic therapy after ascites diagnosis was independently associated with survival (HR: 0.35, 95% CI 0.20-0.61, *P* < 0.001) with a median OS of 62 days (95% CI 51-129 days, *P* < 0.001) versus 16 days (95% CI 11-24 days), respectively.

**Conclusions:**

Liver and peritoneal metastases as well as portal vein obstruction were found to be prognostic factors after ascites diagnosis in mPC patients. Continuation of systemic therapy after ascites diagnosis was associated with a longer OS, which needs to be evaluated in larger clinical trials including quality-of-life assessment.

## Highlights

• Ascites development represents an end-stage condition in mPC patients.
• Liver and peritoneal metastases as well as portal vein obstruction are prognosticators for survival in patients with ascites.
• Continued systemic therapy may improve outcome in these patients compared with best supportive care alone.

## Introduction

Metastatic pancreatic cancer (mPC) is still associated with a limited prognosis of <1 year.^1^^,^^2^ Within this short course of disease patients often suffer from a tremendously high symptom burden directly affecting quality of life (QoL).^3^ In line with that, many patients present with an impaired performance status not only at a later stage, but often at diagnosis already, which limits systemic therapy options. Besides established systemic chemotherapy, supportive treatment approaches therefore play a major role in terms of symptom relief in this distinct patient population.^4^

Ascites represents one of the most frequent symptoms with about 20% of patients being affected.^5^ Multiple risk factors such as liver and peritoneal metastases, liver function, portal vein obstruction (PVO) and inflammation have been described recently as important risk factors increasing the likelihood of ascites in mPC patients.^5^ Ascites development is associated with a considerable symptomatic burden reducing patients mental and physical health, which further hampers QoL in a patient collective with an already dismal prognosis.^6^, ^7^, ^8^ Moreover, it represents a condition which is challenging to treat.^7^^,^^9^ Antitumoral treatment of the underlying cause is often impeded by the patients performance status. Therefore, symptomatic therapy comprising paracentesis and permanent drainages remain the most frequently applied treatment options.^10^ Considering the limited prognosis of a median of 1 month from ascites diagnosis in these patients, identification of prognostic biomarkers at this stage seems crucial to improve QoL.^5^^,^^6^^,^^8^

Therefore, we carried out a structured analysis of clinical factors and their association with survival in a large, real-life cohort of patients with mPC and ascites. We especially focused on potential benefits of chemotherapy continuation after ascites diagnosis in this distinct patient cohort.

## Materials and methods

### Patients

Clinical data including demographics, case history and survival of patients with mPC were collected by retrospective chart review. Patients who developed ascites concomitant to or after diagnosis of metastatic disease were exclusively included in this study. Patients were treated in line with current treatment guidelines and best clinical practice throughout their course of disease at our tertiary care center.^11^ This study was approved by the Ethics Committee of the Medical University of Vienna (vote number 2026 of 2021) and carried out according to the Declaration of Helsinki and its amendments.

### Study design and objectives

As the primary objective we aimed to assess different clinical factors at ascites diagnosis influencing survival. Ascites was predefined as clinically apparent accumulation of intraperitoneal fluid diagnosed either by computed tomography (CT) scan or ultrasound of the abdomen. Patients with an isolated perihepatic ascites formation were excluded from this analysis.

The association of the following factors with survival has been primarily investigated. Laboratory parameters have been grouped in ‘below normal’, ‘normal’ and ‘above normal’ according to reference intervals, which are defined as follows:

• General risk factors: age, sex, Eastern Cooperative Oncology Group performance status (ECOG PS)
• Metastatic sites: liver, peritoneum, lung, bone
• PVO: by thrombosis or tumor formation as diagnosed in CT scans by radiologists
• Parameters of liver function: total protein (64-83 g/l), albumin (35-52 g/l), albumin-bilirubin (ALBI) score. The ALBI score was used as a parameter of liver function because of its association with survival in patients with chronic liver disease and hepatocellular carcinoma (HCC).^12^ The predefined grades, ranging from good (grade 1) to bad prognosis (grade 3) were calculated using serum albumin and bilirubin levels.
• Parameters of systemic inflammation: c-reactive protein (CRP <0.5 mg/dl), platelet-lymphocyte ratio (PLR), neutrophil-lymphocyte ratio (NLR), monocyte-lymphocyte ratio (MLR), leukocyte-lymphocyte ratio (LLR)
• Treatment after ascites diagnosis: best supportive care (BSC), systemic chemotherapy (at least one cycle of chemotherapy administered after ascites diagnosis)

### Statistical analysis

Statistical analysis was carried out using R version 4.2.2. Categorial variables were summarized using absolute counts and percentages, continuous variables were presented as median and range. The aforementioned variables were assessed for their association with survival after ascites diagnosis using a risk regression model. To correct for potential confounders, we additionally included the factors ‘time interval from diagnosis of metastatic disease to ascites diagnosis’ as well as ‘number of applied systemic treatment lines before ascites diagnosis into the risk regression model’. Clinically relevant factors or factors with *P* < 0.1 were entered into a multivariate analysis. A two-sided *P* value of <0.05 was considered a significance threshold and was presented with a 95% confidence interval (CI). Time to ascites was defined as the interval from diagnosis of mPC until ascites development. Overall survival (OS) was defined as interval from first diagnosis of ascites until death or last date of follow-up and estimated with the Kaplan–Meier product limit method. Due to the exploratory and hypothesis-generating design of the present study, no adjustment for multiple testing was applied.^13^

## Results

### Patients’ characteristics

Eight hundred twenty-four patients with mPC treated between 2010 and 2019 at the Medical University of Vienna were identified from the pancreatic cancer database of the Medical University of Vienna and assessed for ascites development. We excluded 241/822 patients (29.3%) due to incomplete data on the clinical course of disease. Among 581 mPC patients with sufficient data, 459 (79.0%) never developed ascites after 414/459 (90.2%) survival events. Among the 122 patients with ascites, 5 patients (4.1%) developed ascites before diagnosis of the malignancy and were therefore excluded as well. Finally, 117 patients developed ascites concomitant to or after mPC diagnosis, representing the final cohort for this analysis.

Among 117 patients, 70 (59.8%) were male and 47 (40.2%) were female. At diagnosis of ascites, patients were a median of 63 years (range: 36-82 years) of age and developed ascites a median of 8.8 months (range 8.4-10.4 months) after initial diagnosis of metastatic disease. Ascites was diagnosed concomitantly to mPC in 4/117 patients (3.4%) and occurred later during the course of disease in 103/117 patients (88.0%). At ascites diagnosis, patients presented with a median ECOG PS of 2 (range 0-4) and had liver metastases in 93/117 (79.5%), peritoneal metastases in 75/117 (64.1%), lung metastases in 40/117 (34.2%) and bone metastases in 9/117 (7.7%) cases. At ascites diagnosis, 73/117 patients (62.4%) were treated with BSC and 44/117 patients (37.6%) by systemic chemotherapy, which was continued in 36/117 patients (30.8%) after ascites diagnosis. Median OS after ascites diagnosis was 27.4 days (range 21.3-42.6 days). Detailed patients’ characteristics at ascites diagnosis are displayed in Table 1.

**Table 1:** Patients’ characteristics at diagnosis of ascites.

Characteristics	At ascites diagnosis
Patients, *N*	117
Sex, *n* (%)	
Female	47 (40.2)
Male	70 (59.8)
Median age, years (range)	63 (36-82)
Median ECOG PS (range)	2 (0-4)
ECOG 0-1, *n* (%)	47 (40.2)
ECOG ≥2, *n* (%)	70 (59.8)
Metastatic sites, *n* (%)	
Liver	93 (79.5)
Lung	40 (34.2)
Peritoneum	75 (64.1)
Bone	9 (7.7)
Median number of metastatic sites (range)	2 (0-4)
Metachronous versus synchronous metastatic disease, *n* (%)	
Metachronous	47 (40.2)
Synchronous	70 (59.8)
Previously applied treatment, *n* (%)	
Surgery of the primary tumor	21 (23.9)
Radiation of the primary tumor	17 (14.8)
Median lines of systemic therapies (range)	2 (1-6)
Occurrence period of ascites, *n* (%)	
At diagnosis of metastatic disease	14 (11.9)
Later during course of disease	103 (88.0)
Treatment at ascites diagnosis, *n* (%)	
Best supportive care	73 (62.4)
Systemic therapy	44 (37.6)
Systemic therapy continued after ascites diagnosis, *n* (%)	36 (30.8)
Applied chemotherapy regimen after ascites diagnosis, *n* (%)	
5-FU + (liposomal) irinotecan	9 (25)
5-FU + oxaliplatin	4 (11.1)
FOLFIRINOX	3 (8.3)
Gemcitabine + nab-paclitaxel	16 (44.4)
Gemcitabine + others	3 (8.3)
Capecitabine alone	1 (2.8)

5-FU, 5-fluorouracil; ECOG PS, Eastern Cooperative Oncology Group performance status.

### Factors associated with survival after ascites diagnosis

#### General risk factors, metastatic sites and PVO

General risk factors associated with a shorter OS according to univariate analysis were ECOG PS [ECOG PS 2 (HR: 2.59, 95% CI 1.20-5.581, *P* = 0.015); ECOG PS 3 (HR: 3.37, 95% CI 1.48-7.67, *P* = 0.004); ECOG PS 4 (HR: 7.63, 95% CI 1.56-37.24, *P* = 0.012)], liver metastases (HR: 1.78, 95% CI 1.1-2.87, *P* = 0.02), peritoneal carcinomatosis (HR: 1.46, 95% CI: 0.99-2.15, *P* = 0.055) and PVO (HR: 3.63, 95% CI 2.06-6.4, *P* < 0.001). Within multivariate analysis, liver metastases (HR: 2.07, 95% CI 1.13-3.79, *P* = 0.018), peritoneal carcinomatosis (HR: 1.74, 95% CI 1.11-2.71, *P* = 0.015) and presence of PVO at ascites diagnosis (HR: 2.52, 95% CI 1.29-4.90, *P* = 0.007) remained independently associated with OS.

#### Liver function and systemic inflammation

An albumin level below normal (HR: 1.22, 95% CI 1.12-3.63, *P* = 0.020) and an ALBI score grade 3 (HR: 2.83, 95% CI 1.09-7.33, *P* = 0.032) were significantly associated with OS according to univariate analysis. None of these parameters remained independently associated with survival according to multivariate analysis. None of the tested parameters of systemic inflammation were associated with survival within univariate analysis.

#### Treatment after ascites diagnosis

**Figure 1:**
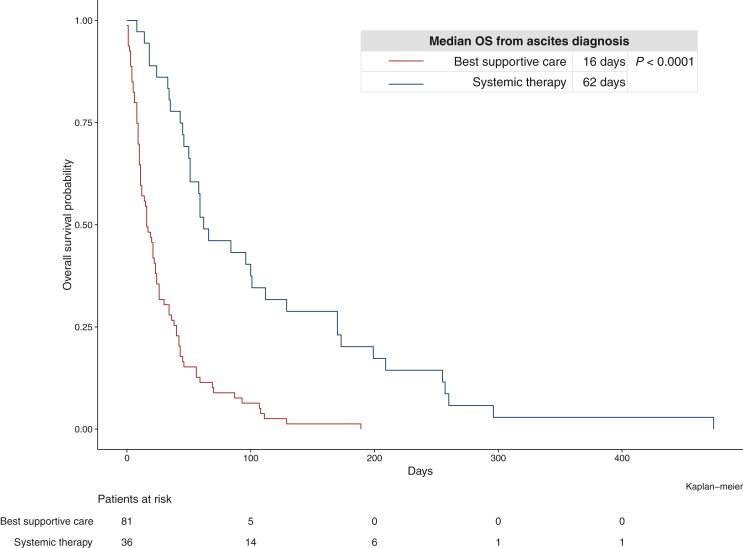
Kaplan–Meier curves of survival after ascites diagnosis according to systemic chemotherapy compared with best supportive care. OS, overall survival.

Systemic chemotherapy after ascites diagnosis was associated with a significantly longer median OS compared with BSC alone according to univariate analysis (HR: 0.27, 95% CI 0.17-0.43, *P* < 0.001), which remained significant (HR: 0.35, 95% CI 0.20-0.61, *P* < 0.001) when corrected for potential confounders from univariate analysis within multivariate analysis such as ECOG PS (*P* > 0.05), metastatic sites (liver: HR: 2.07, 95% CI 1.13-3.79, *P* = 0.018, peritoneum: HR: 1.74, 95% CI 1.11-2.71, *P* = 0.015), PVO (HR: 2.52, 95% CI 1.29-4.90, *P* = 0.007), albumin (*P* > 0.05) or the ALBI score (*P* > 0.05)*.* The factors ‘time from mPC to ascites’ as well as ‘number of applied systemic treatment lines before ascites’ were also entered into the univariate model to correct for additional confounders of the effect of systemic therapy, but were not significantly associated with OS (*P* > 0.1) and therefore not considered in the multivariate model. Patients with a better ECOG PS, however, were more likely to receive systemic therapy (*P* < 0.001). Median OS of patients with systemic chemotherapy after ascites was 62 days compared with 16 days with BSC (*P* < 0.001, log-rank test, Figure 1)*.* Additionally, patients receiving systemic chemotherapy showed an increased rate of remissions of ascites (three patients versus no patients; *P* < 0.001).

**Figure 2:**
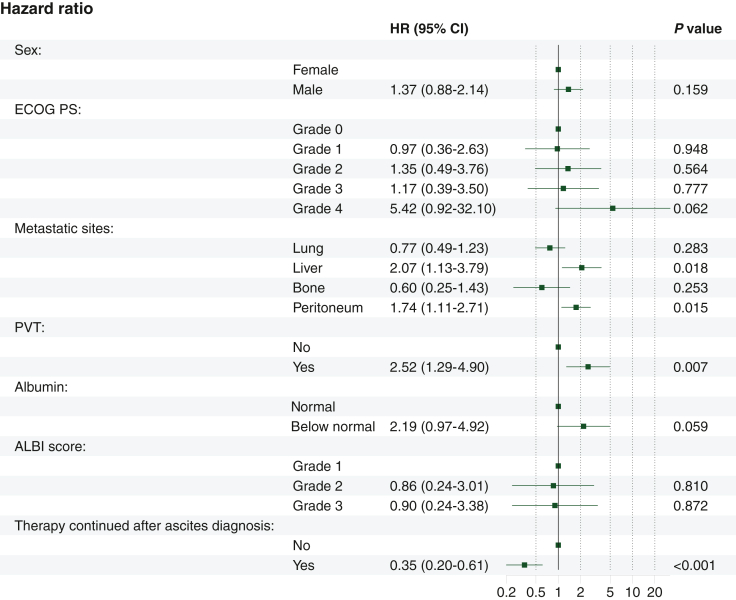
Forest plot of factors associated with survival after ascites diagnosis as stratified by cox proportional hazard models ALBI score, albumin-bilirubin score; CI, confidence interval; ECOG PS, Eastern Cooperative Oncology Group performance status; HR, hazard ratio; PVT, portal vein thrombosis.

Details on the results of the risk analysis are listed in Table 2 and results of the multivariate analysis are displayed in Figure 2.

**Table 2:** Factors associated with survival after ascites diagnosis. Univariate and multivariate analysis stratified by cox proportional hazard models.

	Univariate analysis		Multivariate analysis	
	HR (95% CI)	*P*	HR (95% CI)	*P*
Age at diagnosis of mPC				
(per decade increase)	1.00 (0.99-1.03)	0.466		
Sex				
Female	1			
Male	1.32 (0.90-1.93)	0.156	1.37 (0.88-2.14)	0.159
ECOG PS				
0	1		1	
1	1.28 (0.59-2.78)	0.526	0.97 (0.36-2.63)	0.948
2	2.59 (1.20-5.58)	**0.015**	1.35 (0.49-3.76)	0.564
3	3.37 (1.48-7.67)	**0.004**	1.17 (0.39-3.50)	0.777
4	7.63 (1.56-37.24)	**0.012**	5.42 (0.92-32.1)	0.062
Metastatic sites				
Lung	0.78 (0.53-1.16)	0.216	0.77 (0.49-1.23)	0.283
Bone	1.41 (0.68-2.92)	0.360	0.60 (0.25-1.43)	0.253
Liver	1.78 (1.10-2.87)	**0.019**	2.07 (1.13-3.79)	**0.018**
Peritoneum	1.46 (0.99-2.15)	0.055	1.74 (1.11-2.71)	**0.015**
Portal vein obstruction				
Absent	1			
Present	3.64 (2.06-6.43)	**<0.001**	2.52 (1.29-4.90)	**0.007**
Total protein				
Normal	1			
Below normal	1.29 (0.78-2.14)	0.316		
Albumin				
Normal	1		1	
Below normal	1.22 (1.12-3.63)	**0.020**	2.19 (0.97-4.92)	0.059
ALBI score				
Grade 1	1		1	
Grade 2	1.67 (0.65-4.25)	0.285	0.86 (0.24-3.01)	0.810
Grade 3	2.83 (1.09-7.33)	**0.032**	0.90 (0.24-3.38)	0.872
CRP				
Normal	1			
Above normal	1.49 (0.21-10.70)	0.694		
NLR				
(per unit increase)	1.01 (0.98-1.05)	0.388		
LLR				
(per unit increase)	1.00 (0.99-1.00)	0.676		
MLR				
(per unit increase)	1.10 (0.92-1.32)	0.285		
PLR				
(per 100 unit increase)	1.00 (1.00-1.00)	0.498		
Treatment after ascites				
Best supportive care	1			
Systemic therapy	0.27 (0.17-0.43)	**<0.001**	0.35 (0.20-0.61)	**<0.001**
Duration between metastases diagnosis and ascites				
(per day)	1.00 (1.00-1.00)	0.5		
Lines of systemic therapy				
(per line)	1.01 (0.87-1.18)	0.890		

Significant values are indicated in bold.

ALBI, albumin-bilirubin score; IC, confidence interval; CRP, c-reactive protein; ECOG PS, Eastern Cooperative Oncology Group; HR, hazard ratio; LLR, leukocyte-lymphocyte ratio; mPC, metastatic pancreatic cancer; MLR, monocyte-lymphocyte ratio; NLR, neutrophil-lymphocyte ratio; PLR, platelet-lymphocyte ratio.

## Discussion

Ascites causes considerable discomfort for patients, which impacts their physical and mental well-being and is linked to a dismal prognosis. After our precedent work providing a comprehensive risk factor assessment for the development of ascites in mPC patients,^5^ we now focused on the identification of prognostic biomarkers in this patient collective with a special emphasis on the question of continuation of systemic chemotherapy after ascites diagnosis. The identification of patients at risk for adverse outcomes after ascites diagnosis thereby seems crucial to identify the most suitable treatment option for each individual, particularly considering that this condition represents an end-stage event in pancreatic cancer with a median OS of 27.4 days.

In this study, liver and peritoneal metastases were independently associated with short survival after ascites diagnosis. As published previously, both metastatic sites are common in patients with ascites and were linked to ascites formation causing lymphatic vessel obstruction and increased vascular permeability, as well as portal hypertension and a limited functional liver reserve.^5^^,^^14^, ^15^, ^16^, ^17^ Interestingly, liver metastases were shown to be a stronger prognosticator for OS (HR 2.07) compared with peritoneal carcinomatosis (HR 1.74) once ascites has been diagnosed, whereas the latter seems to play a more important role in the development of ascites. This is well in line with previous reports comprising patients with different malignancies linking liver metastases with especially poor outcomes compared with extrahepatic metastatic sites.^18^^,^^19^ In colorectal cancer, for instance, where patients present with liver metastases in up to 60%, liver involvement represents one of the strongest prognosticators of survival which is further influenced only to a smaller extend by the co-occurrence of other metastatic sites.^20^ One explanation why liver metastases correlate with prognosis more prominently might be the associated impaired organ function leading to a higher rate of complications such as bleedings, cholestasis, encephalopathy, renal insufficiency, hypotension and hypoglycemia. Moreover, liver insufficiency limits the possibility to apply systemic chemotherapy in these patients.^21^ Peritoneal metastases, however, primarily lead to QoL-affecting symptoms such as abdominal pain and dyspnea and influence organ function only in the second place by an increased abdominal pressure.^22^^,^^23^

PVO acted as an independent and highly influential factor (HR 2.52) impeding survival after ascites diagnosis in our study. PVO can be caused by thrombosis, tumor infiltration or compression of the vein by a mass effect, leading to ascites development, portal hypertension and reduced liver function.^5^^,^^24^^,^^25^ A comparable association between PVO and OS could be so far observed in patients with HCC.^26^ Evidence and guidelines for anticoagulation therapy, however, so far exist only for patients with non-malignant liver cirrhosis.^27^ Considering the observed impact of PVO on survival, prospective studies on that topic are highly warranted to provide evidence-based, comprehensive care.

Formerly described independent associations between systemic inflammation and survival could not be verified in this cohort of mPC patients with ascites.^1^^,^^28^^,^^29^ This seems interesting, since parameters such as CRP and NLR have been evaluated thoroughly and their elevation has been linked to impeded survival in other tumor entities.^30^ Similarly, no independent associations between parameters of liver function and survival could be detected, even though decreased albumin levels and an ABLI score of 3 showed correlations in univariate analysis. Hypalbuminemia so far has been reported to be linked to poorer survival with clinical implications in its use in several prognostic scores such as the Glasgow Prognostic Score for systemic inflammation or the ALBI score as validated liver function score.^31^^,^^32^ The here investigated ALBI score was even shown to be associated with survival in HCC.^33^ As neither systemic inflammation nor liver function parameters were independently associated with survival after ascites diagnosis, however, we hypothesize that potential effects of these parameters may not be detected due to short survival times after ascites diagnosis in patients with mPC.

Another interesting finding of this present study was that continuation of systemic chemotherapy compared with BSC alone was associated with an improved outcome (HR 0.34) with a difference of 46 days and therefore could be identified as the only positive prognosticator of survival. This effect was independent of previously applied therapy lines and time from diagnosis of mPC to ascites, which seems even more intriguing since the majority was pretreated with at least two systemic therapy lines and developed ascites rather late after 8.9 months. Additionally, an impaired performance status of patients did not change this observation. Therefore, some patients might indeed benefit from continuation of chemotherapy, even though they have to be selected very carefully considering its impact on QoL. We indeed observed three cases of complete ascites remission under systemic chemotherapy, however, even though the sample size for this analysis was rather small. As most patients have undergone several treatment lines before being diagnosed with ascites, selecting an appropriate drug certainly might be challenging as the most effective agents have been exhausted previously. Another factor that needs to be taken into account is its tolerability, which can be limited at late stages.^11^ With the accumulation of fluid in the peritoneal cavity, pharmacokinetics might change and should be taken into account. Some agents may not be able to penetrate into the ascitic compartment, thereby limiting its effects on peritoneal metastases.^34^^,^^35^ So far, there are no prospective studies evaluating which systemic treatment would be most appropriate to apply, leaving the drug choice entirely to the clinician’s experience. Still, if there are effective treatment options available and a good performance status present, systemic therapy might be considered based on current treatment guidelines.^21^ Further prospective studies including QoL assessment are clearly warranted to better identify patients who might benefit from systemic treatment at this stage. As data indicate that early palliative care not only improves QoL, but also the outcome of patients, supportive therapy should be a key component of treatment in any case.^36^, ^37^, ^38^

The most important limitation of this study is the retrospective design, which did not allow for standardized ascites evaluation or randomization to different systemic therapies after ascites. Also, preexisting liver diseases and their impact on survival therefore could not be considered unfortunately. The effect of systemic chemotherapy on QoL would have been of major interest, which unfortunately also was not possible to assess. Comparison of different chemotherapy regimens could not be carried out due to small sample sizes and varying time points of their application. This study, however, is the first and so far largest structured analysis of factors associated with outcomes in mPC patients after ascites diagnosis as well as investigation of systemic therapy continuation in this setting, which hopefully encourages further research efforts in this field.

In conclusion, this analysis identified several clinical factors linked to impeded survival after ascites diagnosis in mPC patients. The continuation of systemic therapy thereafter was the only positive prognosticator we identified.
